# Bacterial Community Associated with Organs of Shallow Hydrothermal Vent Crab *Xenograpsus testudinatus* near Kuishan Island, Taiwan

**DOI:** 10.1371/journal.pone.0150597

**Published:** 2016-03-02

**Authors:** Shan-Hua Yang, Pei-Wen Chiang, Tin-Chang Hsu, Shuh-Ji Kao, Sen-Lin Tang

**Affiliations:** 1 Biodiversity Research Center, Academia Sinica, Taipei, Taiwan; 2 Molecular and Biological Agricultural Sciences, Taiwan International Graduate Program, Academia Sinica, Taipei, Taiwan; 3 Graduate Institute of Biotechnology and Department of Life Sciences, National Chung-Hsing University, Taichung, Taiwan; 4 Research Center for Environmental Changes, Academia Sinica, Taipei, Taiwan; 5 State Key Laboratory of Marine Environment, Xiamen University, Fujian, China; 6 Biotechnology Center, National Chung-Hsing University, Taichung, Taiwan; National Cheng-Kung University, TAIWAN

## Abstract

Shallow-water hydrothermal vents off Kueishan Island (northeastern Taiwan) provide a unique, sulfur-rich, highly acidic (pH 1.75–4.6) and variable-temperature environment. In this species-poor habitat, the crab *Xenograpsus testudinatus* is dominant, as it mainly feeds on zooplankton killed by sulfurous plumes. In this study, 16S ribosomal RNA gene amplicon pyrosequencing was used to investigate diversity and composition of bacteria residing in digestive gland, gill, stomach, heart, and mid-gut of *X*. *testudinatus*, as well as in surrounding seawater. Dominant bacteria were Gamma- and Epsilonproteobacteria that might be capable of autotrophic growth by oxidizing reduced sulfur compounds and are usually resident in deep-sea hydrothermal systems. Dominant bacterial OTUs in *X*. *testudinatus* had both host and potential organ specificities, consistent with a potential trophic symbiotic relationship (nutrient transfer between host and bacteria). We inferred that versatile ways to obtain nutrients may provide an adaptive advantage for *X*. *testudinatus* in this demanding environment. To our knowledge, this is the first study of bacterial communities in various organs/tissues of a crustacean in a shallow-water hydrothermal system, and as such, may be a convenient animal model for studying these systems.

## Introduction

A deep-sea hydrothermal vent is one of the most extreme environments on earth, due to its poorly oxygenated, oligotrophic and toxic ecosystem [1]. In such ecosystems, chemolithotrophic bacteria are common residents [2]. Bacteria associated with host animals (e.g., Crustacea), are believed to support their hosts and enable them to adapt to their extreme environment, including high toxicity and limited nutrients [3–4].

Some chemolithotrophic bacteria have been identified and characterized in deep-sea hydrothermal vent shrimp *Rimicaris exoculata* [5–7] and in crabs, including *Kiwa* spp. [8–9] and *Shinkaia crosnieri* [10]. In addition, some Crustacea-associated bacteria in hydrothermal vents not only have host specificity, but also site specificity within the host’s gut or gill chamber [5–7], consistent with important roles in nutrient supply [11] and detoxification [6] for their hosts, which live in oligotrophic and toxic hydrothermal vent environments [5,12].

Shallow-water hydrothermal vents are usually near active coastal or submarine volcanoes and also provide an oligotrophic and toxic environment for animals and microorganisms [1]. The crab *Xenograpsus testudinatus* predominates in shallow-water, sulfur-rich/highly acidic hydrothermal (pH 1.75–4.6) [13] vents near Kueishan Island, northeastern Taiwan. This crab is one of the few known vent-endemic species at depths < 200 m [13]. Unlike the deep-sea hydrothermal system, there is no chemolithoautotrophic food-web in the shallow-water hydrothermal vent off Kueishan [13]. Because the biodiversity of shallow-water hydrothermal vent is relatively low compared to deep-sea hydrothermal vents, *X*. *testudinatus* has a unique opportunistic feeding style; when the current is weak, they eat zooplankton killed by the vent’s sulfurous plumes [13]. Despite some recent studies regarding nutrient acquisition by *X*. *testudinatus*, there are apparently few reports characterizing *X*. *testudinatus*-associated bacteria.

Due to differences in food-webs between shallow-water and deep-sea hydrothermal systems, Crustacea-associated bacteria in a shallow-water thermal vent might differ from those in a deep-sea hydrothermal vent. Therefore, the objective was to characterize the bacterial community in organs of *X*. *testudinatus* in the vicinity of sulphur-rich hydrothermal vents in shallow water near Taiwan.

## Materials and Methods

### Sampling site and sample collection

The sampling site was located near Kueishan Island (121°57’E, 24°50’N), Taiwan. The dominant species *Xenograpsus testudinatus* and seawater samples were collected at the hydrothermal venting area, approximately 8–20 m from the island [13]. The surface of *X*. *testudinatus* is covered by a filamentous biofilm [14]. Two crabs (one male and one female) were collected (SCUBA-diving) in depths ranging from 10 to 15 m, in May 2009. After sampling, crabs were kept in an aerated cooling box, directly transported to the laboratory, and frozen at -20°C before assaying (interval from collection to freezing was < 12 h). A seawater sample (50 ml) was also collected from the sampling place of the shallow-water hydrothermal vent. The target species *X*. *testudinatus* in this study is not listed as endangered or protected and was not collected from national parks or natural reserves in Taiwan, thus no specific permission was required for sampling.

### Total DNA extraction

**Fig 1:**
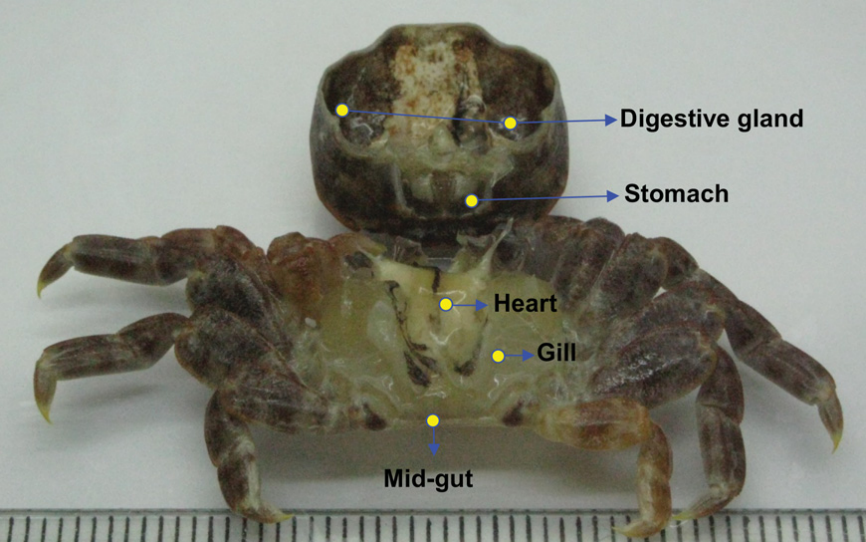
*Xenograpsus testudinatus* and organs. Organs selected for study are indicated (scale = mm).

The entire dissection procedure was performed in a biological safety cabinet. Before dissection, crabs were washed twice with sterile seawater. All dissection instruments were sterilized over an open flame to eliminate residual DNA and washed with 75% ETOH to prevent cross-contamination. After removing upper carapaces, the digestive gland, gill, stomach, heart and mid-gut from each crab were excised for DNA extraction (Fig 1). Total genomic DNA was extracted according to a modified standard phenol–chloroform procedure incorporating a grinding step in liquid nitrogen to mechanically lyse cells [15]. After extraction, DNA samples of various body parts were denoted as D (digestive gland), S (stomach), H (heart), G (gill), and M (mid-gut). Bacterioplankton from seawater samples were filtered on cellulose acetate membranes (pore size, 0.2 μm; ADVANTEC, Tokyo, Japan). Microbial biomass was removed from membranes by washing with TE buffer (50 mM Tris-HCl, 1 mM EDTA, pH 8.0). Suspensions were collected in microtubes and total DNA was extracted by the same method as described. The DNA from seawater samples was denoted as SW. All DNA samples were stored at -20°C.

### Amplification of V6-V8 hypervariable regions of 16S rRNA gene

The 16S ribosomal RNA gene was amplified by PCR, using a pair of bacterial universal primers: U968F (5’-AACGCGAAGAACCTTAC-3’) and U1391R (5’-ACGGGCGGTGWGTRC-3’), specially designed for the bacterial V6-V8 hypervariable region [16–17]. The total volume was 50 μl, including 4 μL of 2.5 mM dNTP, 1 μL of each primer (10 μM), 0.5 μL of 5U TaKaRa Ex Taq HS (Takara Bio, Otsu, Japan), 5 μL of 10x Ex Taq buffer and 5 μL (10–20 ng) template DNA. The amplification program was conducted using a PxE Thermal Cycler (Thermo Electron Corporation, Milford, MA, USA). The first cycle was initiated at 94°C for 5 min, and followed by 30 cycles; each cycle was 94°C for 30 s, 52°C for 20 s, and 72°C for 45 s. The last cycle was 72°C for 10 min, before cooling at 4°C. Thereafter, PCR products were separated (electrophoresis) and visualized with a UV trans-illuminator (ImageQuant 300, GE Healthcare, Piscataway, NJ, USA).

### DNA tagging PCR and massively parallel pyrosequencing

The DNA tagging PCR (five cycles) was used to tag 5′ ends of each V6-V8 amplicon [16]. For this, DNA amplified from the same parts of male and female crabs were pooled equally into a sample to provide a comprehensive bacterial community survey and ignore gender variation. Pooled lots of each tagged V6-V8 DNA samples (final DNA content ~ 0.5 μg) were sent to Mission Biotech (Taipei, Taiwan) for massively parallel pyrosequencing using a Roche 454 GS FLX Titanium System. A total of 37157 sequences were generated and sorted. Novel bacterial community sequences were deposited in GeneBank (SRA accession PRJNA296845).

### Data analysis

After trimming, chimerical reads were detected by UCHIME (http://drive5.com/uchime) [18]. For operational taxonomic units (OTU) analysis, quality-filtered reads were pooled together and analyzed with the UPARSE pipeline [19] except the chimera removal step. In UPARSE, de-replication was done with the options “–derep_prefix” and “–minsize 2” and OTUs were generated at 97% identity. Each OTU was searched (with global alignment) against the Greegenes 13_5 database to find corresponding taxonomy of the best hit using USEARCH. Alignment was calculated using MOTHUR software to define operational taxonomic units (OTUs) with a 3% cut off value in sequence dissimilarity. Defined OTUs were used to estimate the Shannon-Weaver diversity index, Chao1 estimator, and the Simpson index, as well as to construct rarefaction curves, and to calculate evenness and richness of the bacterial community. Unclassified OTUs were manually searched in NCBI using BLASTn.

Relative abundances of each classified bacterial classes in individual samples were incorporated into a matrix, with Bray-Curtis used to estimate a distance matrix. Results were presented in hierarchical clustering (CLUSTER) and non-Metric multi-Dimensional Scaling (nMDS) using Primer 6 software (PRIMER-E, Lutton, Ivybridge, UK) to analyze to relationships between bacterial communities among various samples.

### Quantitative PCR

For the quantitative PCR assay, two primer sets were designed to target the dominant group OTU1 and OTU7, which were representative of Epsilonproteobacteria and Gammaproteobaceria respectively, by using Primer-BLAST tool on BLAST search (NCBI): Episilon-F (5'-CTCGTGTCGTGAGATGTTGG-3') and Episilon-R (5'-GCGAAGGCAGTCTCACTAGA-3'), Gamma-F (5'-TGTTGGGTTAAGTCCCGCAA-3') and Gamma-R (5'-CACCTTCCTCCGGTTTGTCA-3'). The hypervariable V6 region for 16S rRNA gene was used as an endogenous control, using a pair of primers: 967F (5'-CAACGCGAAGAACCTTACC-3') and 1046R (5'-CGACAGCCATGCANCACCT-3') [16]. Quantitative PCR reaction was performed in an ABI 7300 Real-Time PCR system. The PCR program consisted of 2 min at 95°C, 45 cycles of 15 s at 95°C, and followed by 30 s at 60°C, using Platinum SYBR Green qPCR SuperMix-UDG (Invitrogen, Waltham, MA, USA). A dissociation stage was performed to confirm the specificity of the product and to avoid the production of primer dimers; the parameters consisted of 15 s at 95°C, 1 min at 60°C, 15 s at 95°C, and 15 s at 60°C. For all reactions, 2μl (10 ng) of template DNA was added to a reaction of 20μl. These reactions contained 10 μl of Platinum SYBR Green qPCR SuperMix-UDG (Invitrogen), 3.8 μl of sterilized nuclease-free water, 0.4 μl each of the forward and reverse primers, 0.4μl of 1X ROX Reference Dye, and 2 μl of DNA template. Each sample was performed in duplicate.

## Results

The V6 to V8 hypervariable regions of bacterial 16S ribosomal RNA genes were surveyed to determine bacterial community composition in various crab body parts/tissues (Fig 1). A total of 37157 sequences were generated; the number of V6-V8 sequences in each sample varied from 5287 to 7253 (Table 1). Rarefaction curves approached a plateau (S1 Fig), which indicated sequences adequately represented bacterial composition in crab samples. Shannon’s index of crab samples ranged from 0.866 to 2.184, whereas the surrounding seawater sample was 3.065. The Chao1 index ranged from 66 to 192 in crab samples and 246 in seawater. There was much lower bacterial diversity in crab than surrounding seawater. Within crabs, Shannon index of bacterial communities was least in the stomach, but greatest in the mid-gut.

**Table 1:** Sequence information and diversity estimates as represented in 16S rRNA gene libraries.^a^

Index	D	G	H	M	S	SW
*S*^b^	54	150	83	118	51	204
Singleton OTU	19	49	27	39	17	52
*N*^c^	5287	5771	6813	5823	7253	6210
Evenness^d^	0.299	0.398	0.268	0.457	0.22	0.576
Richness^e^	4.83	12.76	6.78	10.09	4.14	13.44
Shannon	1.195	1.996	1.185	2.184	0.866	3.065
Chao1	88	192	114	155	66	246
	(64–162)^f^	(170–235)^f^	(95–165)^f^	(134–200)^f^	(55–98)^f^	(225–289)^f^
Ace	74.405	201.497	111.057	156.523	67.951	249.103
Simpson	0.492	0.34	0.585	0.257	0.638	0.137

D = digestive gland; G = gill; H = heart; M mid-gut; S = stomach; SW = seawater.

^a^Calculations based on OTUs formed at an evolutionary distance of ≤0.03 (~97% identity)

^b^*S* = number of OTUs

^c^*N* = number of sequences

^d^Evenness = Shannon/ln (the number of OTUs)

^e^Richness = (number of singleton OTUs-1)/log_10_*N*. Maximum value is (*N*-1)/log_10_*N*.

^f^95% Confidence intervals for Chao 1 estimator are in parentheses.

**Fig 2:**
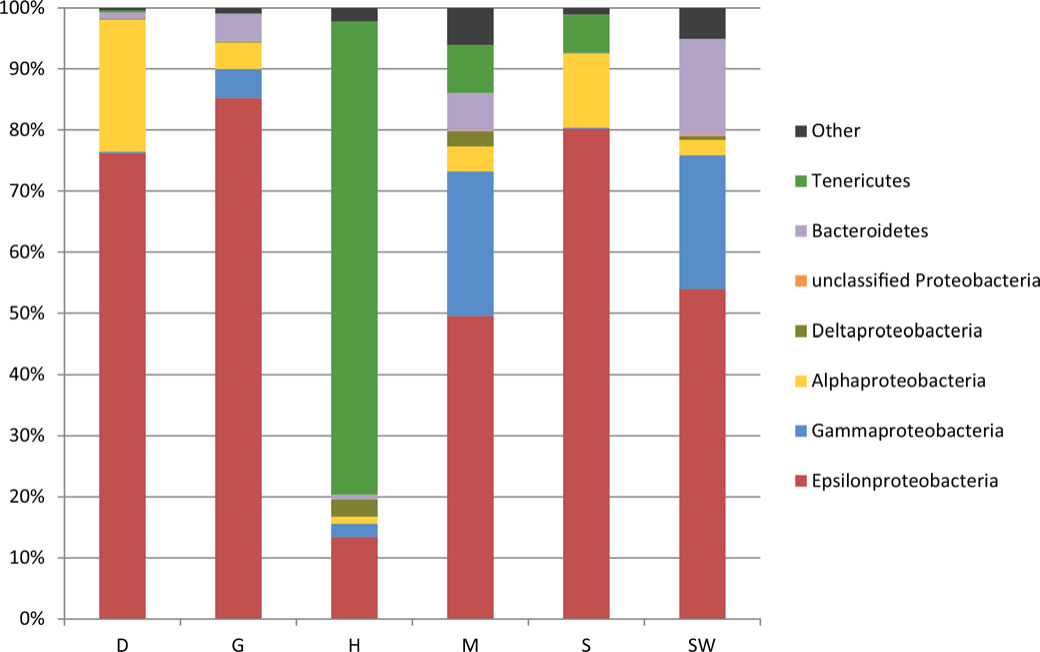
Relative abundance of major bacterial taxa in crab organs and in seawater. The *X*. *testudinatus* related samples are denoted as S (stomach), H (heat), G (gill), M (mid-gut) and D (digestive gland), whereas seawater is SW. Bacterial lineages without identifiable class names are indicated by phylum with N/A.

**Fig 3:**
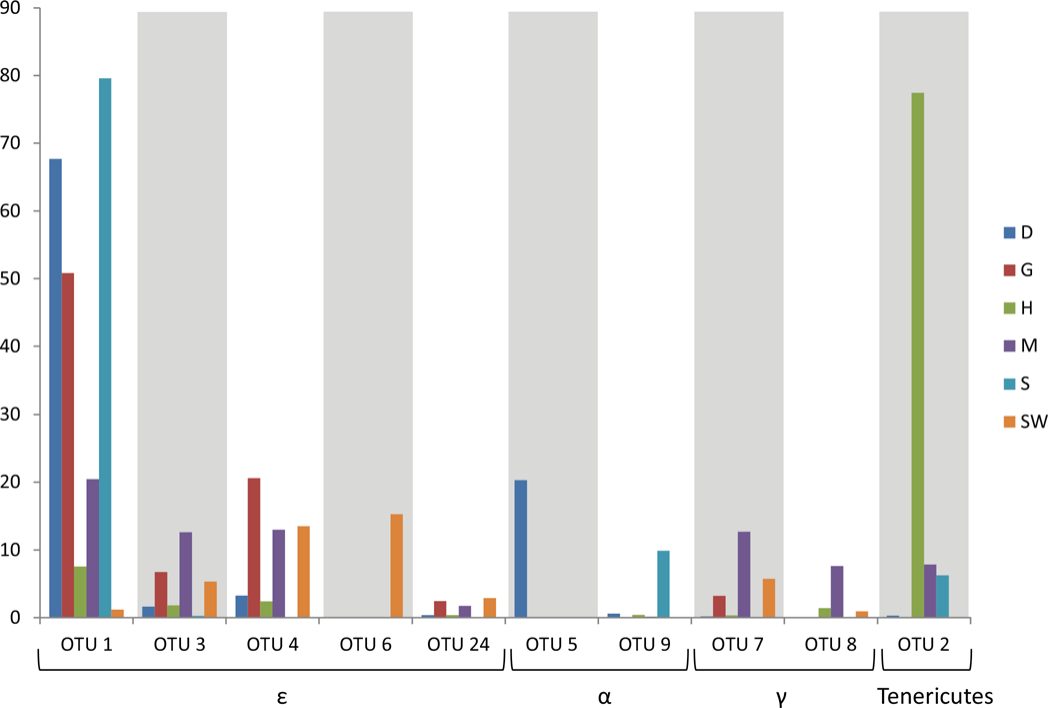
Relative abundances of the top 10 OTUs in the organs of *X*. *testudinatus* and seawater. Taxa of each OTU are shown as α (Alphaproteobacteria), γ (Gammaproteobacteria), ε (Epsilonproteobacteria) and Bacteria (unclassified bacteria). Colors indicate *X*. *testudinatus*-related samples and seawater. OTU5 was present only in D sample; OTU6 and OTU9 were prevailing in SW and S sample, respectively. D = digestive gland; G = gill; H = heart; M = mid-gut; S = stomach; SW = seawater.

**Fig 4:**
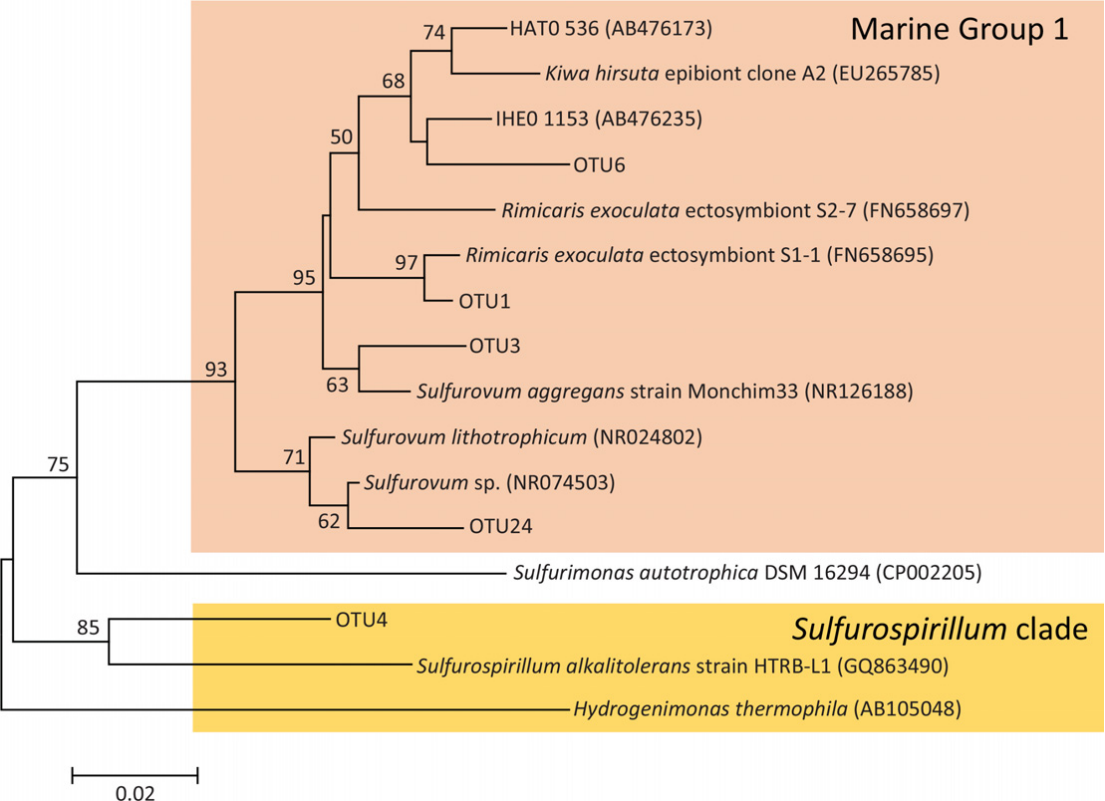
Neighbor-joining tree of Epsilonproteobacteria associated with *X*. *testudinatus* based on 16S rRNA gene sequences. Robustness was tested using 1000 bootstraps for re-sampling of the tree.

At the phylum level, bacterial composition in the seawater and crab were similar, but seawater-associated bacterial communities were more diverse than any sample of crab (Fig 2). Epsilonproteobacteria was the most dominant bacterial class, accounting for 53.94% of total sequences in seawater. In crab samples (excluding heart), Epsilonproteobacteria was also the most dominant bacterial class in stomach (80.18%), gill (85.20%), and digestive gland (76.18%). There were a total of 221 OTUs in the study. Among total OTUs, five of the top 10 OTUs affiliated to Epsilonproteobacteria (Fig 3). Four of these epsilonproteobacterial OTUs (OTU1, 3, 6, and 24) were *Sulfurovum*-related sequences, clustered within Marine Group 1 (Fig 4) [20]. However, only OTU4 was a *Sulfurospirillum*-related sequence (Fig 4). Relative abundance of OTU1 was highest in samples of crab, but relatively low in seawater. In contrast, OTU6, the OTU with the highest relative abundance in the seawater, was relatively low in crab samples. Compared to the most abundant Gammaproteobacteria OTU (OTU7), relative folds of the most abundant Epsilonproteobacteria OTU (OTU1) in each sample obtained by qPCR corresponded to relative abundance of bacterial composition obtained by sequencing, which also supported dominance of Epsilonproteobacteria (S2 Fig). In qPCR, Epsilonproteobacteria was 140.45 and 157.51 times higher than Gammaproteobacteria in stomach and digestive gland, respectively (S2 Fig), and only 1.97 in seawater and 1.08 times in mid-gut. Similarly, in relative abundance of bacterial sequences, Epsilonproteobacteria was 443.23 times higher than Gammaproteobacteria in stomach and 284.21 times higher in digestive gland respectively, but only 2.46 times in seawater and 2.10 times in mid-gut (Fig 2).

Although Gammaproteobacteria was not as dominant as Epsilonproteobacteria, it was dispersed widely in all samples, particularly in seawater (21.94%) and mid-gut (23.65%). However, further comparison revealed distinct differences between crab parts and seawater with regards to Gammaproteobacteria. The dominant gammaproteobacterial OTU (OTU7) was most closely related (99% identity) to the phylotype reported in *R*. *exoculata* [7]. This OTU was dominant in digestive gland, gill, heart, mid-gut, and seawater. Additionally, in the mid-gut, and heart, two OTUs that belonged to *Vibrio* were also dominant. In the stomach, there was limited Gammaproteobacteria, whereas a *Methylomonas*-related sequence was only detected in seawater.

Alphaproteobacteria was also not as dominant as Epsilonproteobacteria, but it was detected in every sample (Fig 2). In particular, OTU5, which was affiliated to Alphaproteobacteria (86% in sequence identity), was only retrieved in the digestive gland, whereas OTU9, which was affiliated to *Roseovarius tolerans* (98% in sequence identity), mainly existed in the stomach of *X*. *testudinatus* (Fig 3).

In the heart sample, OTU2, which was affiliated to the class Mollicutes based on blast against the database at NCBI (sequence identity of 89%), was most dominant (relative abundance of 79.65%; Fig 2). Although OTU2 was also present in stomach, mid-gut and digestive gland, it was present in heart, but never retrieved in seawater (Fig 3).

**Fig 5:**
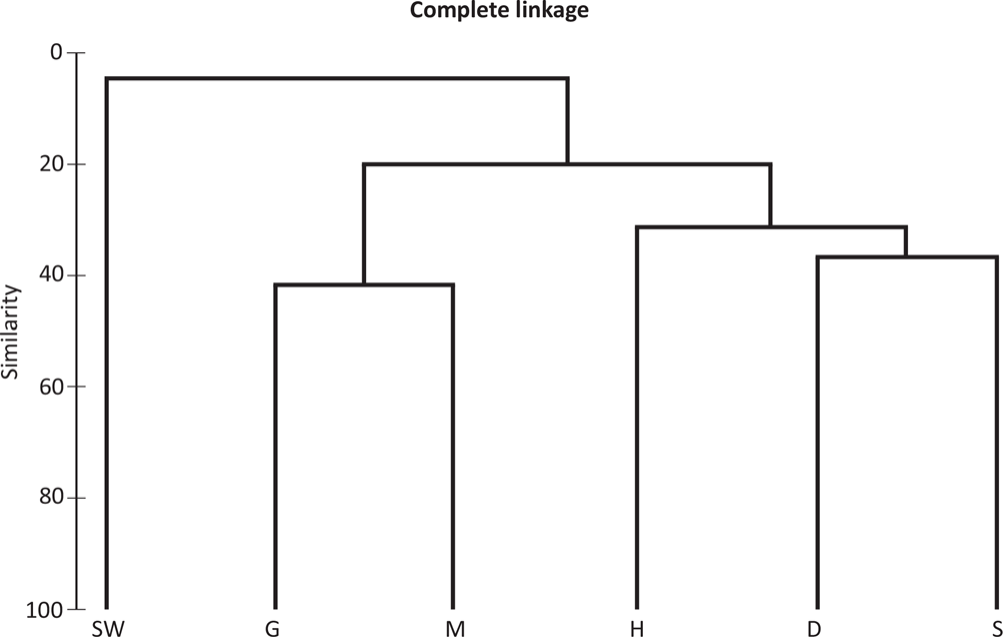
Cluster analysis of organs of *X*. *testudinatus* and seawater. Bray-Curis similarity index was calculated using relative percentage of each class in each sample, and hierarchical clustering was performed in PRIMER 6. The *X*. *testudinatus*-related samples were denoted as S (stomach), H (heat), G (gill), M (mid-gut) and D (digestive gland), whereas seawater is SW.

Based on the clustering result (Fig 5), crab-associated bacterial communities appeared to be clustered together, which was distinctly different from the seawater bacterial community. In addition, bacterial composition of gill and mid-gut were more similar to each other, whereas digestive gland and stomach not sure congregated in clustering analysis.

## Discussion

### *X*. *testudinatus* associated Gamma- and Epsilonproteobacteria

The major bacterial group associated with inside *X*. *testudinatus* was Epsilonproteobacteria, a common bacterial group in hosts of deep sea hydrothermal systems [21]. Since there was much less diversity of bacteria in crabs than in sea water in this study, we inferred that there was potential host specificity of Epsilonproteobacteria in *X*. *testudinatus*. Furthermore, although composition of bacterial classes in both sea water and crab were dominated by Epsilonproteobacteria, the dominant epsilonproteobacterial OTUs in crab and sea water differed. Notably, OTUs of Epsilonproteobacteria had distinct preferences to host and seawater. In this regard, OTU1, which was predominant in *X*. *testudinatus*, was closely related to Epsilonproteobacterum ectosymbiont of deep sea hydrothermal vent shrimp *Rimicaris exoculata* (i.e., 99% in sequence identity); however, OTU6, closely related to genus *Sulfurovum* sp., was only dominant in seawater. Epsilonproteobacteria were widely present in other crustaceans dwelling in hydrothermal vent, including crabs (*Kiwa hirsute* and *Shinkaia crosnieri*) [8, 10] and shrimp (*Rimicaris exoculata*) [20]. A symbiotic relationship between bacterial epibions and *Rimicaris exoculata* has been proposed [5–7], and a mutualistic relationship between *R*. *exoculata* and epibions was reported [11]. Epsilonproteobacteria in *R*. *exoculata* might be capable of autotrophic growth by oxidizing reduced sulfur compounds [11, 20]. In this regard, Epsilonproteobacteria is able to assimilate inorganic carbon and directly transfer nutrients to the host *R*. *exoculata* through the shrimp integument rather than via the digestive tract [11]. In addition to Epsilonproteobacteria, there was also *Thiotrichaceae*, a chemolithoautotrophic sulfur-oxidizing Gammaproteobacteria frequently recovered from various deep-sea invertebrates of hydrothermal environments [9]. In the present study, two *Thiotrichaceae* genera, *Thiothrix* and *Thioploca*, were detected from *X*. *testudinatus*. Based on this study, *X*. *testudinatus*-associated dominant bacteria were similar to bacteria from crustaceans in deep-sea hydrothermal environments.

We inferred that there was some host specificity for the bacterial community in *X*. *testudinatus*, which is highly mobile and can live in either vent or non-vent environments [14]. Unlike deep-sea hydrothermal environments, some upper sublittoral vents are considered mixed photosynthetic-chemosynthetic systems [22]. Based on gut contents, Wang et al. [14] suggested that *X*. *testudinatus* not only feed on the plankton killed by vent plumes [13], but they also eat dead crustacean bodies and detritus. Thus, *X*. *testudinatus* may be a generalist omnivore [14]. Moreover, fatty acid profiles in *X*. *testudinatus* mid-gut glands included high concentrations of vaccenic (18:1(n-7)) and palmitoleic (16:1(n-7)) acids [23]. Therefore, in addition to phytoplankton [13], bacteria could be a nutrient source of *X*. *testudinatus*. Although the feeding strategy of the *X*. *testudinatus* was apparently different from that of crabs or shrimps in deep-sea hydrothermal vents, we inferred that *X*. *testudinatus* associated Epsilonproteobacteria might constitute a nutrient source for *X*. *testudinatus* in this shallow-water hydrothermal system.

The bacterial community might also benefit the host by detoxification of potential metabolic inhibitors in deep-sea hydrothermal vent environments [6]. For example, by converting H_2_S to elemental sulfur by Gamma- and Epsilonproteobacteria, *Rimicaris exoculata* would benefit from access to less toxic products [6]. Water around shallow-water hydrothermal vents in Kueishan Island contains considerable carbon dioxide, nitrogen, oxygen, sulphur dioxide, hydrogen sulphide [24] and other metal ions, including Mg^2+^, Ca^2+^, Fe^2+^, Cu^2+^, Al^3+^ and Mn^3+^ [25], making this a relatively toxic environment for *X*. *testudinatus* and its prey [23]. Therefore, perhaps *X*. *testudinatus* associated Gamma- and Epsilonproteobacteria have an essential role for their host, namely detoxification.

Some Epsilonproteobacteria may be autotrophic denitrifiers in the hydrothermal system [26]. Among the Epsilonproteobacteria in the deep sea hydrothermal environment, *R*. *exoculata* related epibiontic Epsilonproteobacteria have potential to produce ammonium. Therefore, Epsilonproteobacteria might uptake ammonium from the environment or host when they are free living [6]. Perhaps *X*. *testudinatus* associated Epsilonproteobacteria derive benefits from the host, similar to *R*. *exoculata*-associated Epsilonproteobacteria.

### Bacterial diversity and potential organ specificity bacteria in *X*. *testudinatus*

Bacterial diversity in the gill seems to be high, because gill is the first organ in direct contact with water and suspended particles. However, in this study, bacterial diversity in mid-gut was higher than that in gill. One potential reason is accumulation of trace metals in gill; concentrations of dissolved metals may be increased by the acidic environment and high temperature, accumulating in the gill of *X*. *testudinatus* by respiration [27]. Concentrations of Al, Cd, Co, Ni, Cu, and Zn were higher in gill than in exoskeleton, hepatopancreas or muscle [27]. Although the relationship between bacteria and the accumulated metals was not clear, those specific metals might influence composition of bacteria in the gill of *X*. *testudinatus*. In addition, Hu et al. [23] reported that enzymes, including trypsin-like enzymes and proteinases, in the mid-gut glands of *X*. *testudinatus* had significantly greater thermal tolerance. The microenvironment for the activity of these enzymes might also influence bacterial community structure in the mid-gut of the host.

Interestingly, unlike other parts of crabs, the predominant bacterial OTU (OTU2) in the heart was Mollicutes (in the phylum Tenericutes), not Gamma- or Epsilonproteobacteria. The class Mollicutes is classified by an absence of a cell wall. Many Mollicutes are parasites of insects, plants and crustacean [28]. In previous studies, OTUs belong to Mollicutes were present in the gut of hydrothermal shrimp *Rimicaris exoculata* [5] and crab *X*. *testudinatus* [29]. Because this OTU was mainly in the heart, we inferred that this was a different microenvironment from other parts of crab, and also organ specificity for *X*. *testudinatus*-related bacteria. Although it is difficult to get further taxonomic information regarding OTU2 and the potential relationship of these bacteria and host, based on heart-associated bacteria discovered in this study, we speculated that there might be a specific role for these bacteria in *X*. *testudinatus*.

Alphaproteobacterial OTUs were only detected in the stomach or digestive gland of *X*. *testudinatus*. Although further taxonomic information of OTU5 was not readily available, OTU5 which was only present in the digestive gland was close to an alphaproteobacterial sequence from the digestive gland of European shore crab *Carcinus maenas* [30]. Perhaps the digestive gland of *X*. *testudinatus* provided a unique environment for these bacteria. In addition, *Roseobacter* is usually present in macroalgae [31] and marine snow [32]. In this study, OTU9, which prevailed in the stomach of *X*. *testudinatus*, was affiliated to a strain of *Roseovarius tolerans* from macroalgae [33]. Perhaps these were algae- or marine snow-associated bacteria and eaten by the host.

*Vibrio* comprises more than 110 recognized species, of which many are pathogenic to humans and animals [34–35], although some isolates have probiotic properties [36]. Recently, the presence of virulence genes in a deep-sea hydrothermal vent *Vibrio* species was reported [37]. Those virulence genes were homologs of those in *Vibrio* species pathogenic to humans. Thus, it has been suggested that these pathogenicity genes serve a far more basic ecological role than only causation of human disease [37]. For example, in symbiosis between the squid *Euprymna scolopes* and luminous bacterium *Vibrio fischeri* [38], this bacterium helps the host by producing light in the organ, forming counterillumination to avoid predators. In the present study, these OTUs, which affiliated to *Vibrio*, were only present in the heart and mid-gut of *X*. *testudinatus*, and in seawater. Although the specific functions, pathogenicity, or probiotic properties of *X*. *testudinatus* associated *Vibrio* are unknown, the presence of *Vibrio* in the healthy host of shallow-water hydrothermal system in this study might provide impetus for further study regarding evolution of *Vibrio* and its genomic events.

## Conclusions

Chemoautotrophic Gamma- and Epsilonproteobacteria were recovered from several crabs around hydrothermal vents and cold seep habitats. Although roles of Gamma- and Epsilonproteobacteria in *X*. *testudinatus* were not clear, we inferred that in these shallow-water, sulfur-rich/highly acidic hydrothermal vents, *X*. *testudinatus* acquired energy not only based on opportunistic feeding (consuming dead zooplankton killed by vent’s sulfurous plumes), but also from nutrient transfer from probable symbiotic Epsilonproteobacteria and/or Gammaproteobacteria.

Kueishan Island is subjected to the Kuroshio Current, one of the world’s fastest currents [39]. The Kuroshio Current is low in nutrients and plankton populations, but warm with rapid flow, running from tropical Philippines through subtropical Taiwan and Okinawa to the temperate region of Japan, transferring heat from equatorial regions to higher latitudes [40–43]. Thus, due to differences between environments of deep-sea and shallow-water hydrothermal vents, the way to obtain nutrients by crustaceans in shallow-water hydrothermal vent might be more complicated than in deep-sea hydrothermal vents, including chemosynthetic and photosynthetic primary productions and a heterotrophic system. Versatile ways to obtain nutrients may confer an adaptive advantage for the crab *X*. *testudinatus* in the shallow hydrothermal vent, and account for *X*. *testudinatus* being dominant in this environment. In addition, our study revealed a potential symbiotic relationship between *X*. *testudinatus* and *X*. *testudinatus* associated Gamma- and Epsilonproteobacteria, which may be similar to the relationship between deep-sea hydrothermal vent crustaceans and Gamma- and Epsilonproteobacteria bacteria. Therefore, *X*. *testudinatus* may provide a convenient animal model for hydrothermal systems.

## Supporting Information

S1 Fig The rarefaction curve of bacterial 16S rRNA genes in organs of *X*. *testudinatus* and seawater. Operational taxonomic units (OTUs) were defined by a 3% cut off value in sequence dissimilarity. Related samples were denoted as S (stomach), H (heat), G (gill), M (mid-gut), D (digestive gland) and SW (seawater).
(DOCX)

S2 Fig Relative fold change of 16S copy number of Gammaproteobacteria and Epsilonproteobacteria in organs of *X*. *testudinatus*. Relative folds were calculated by the comparative 2^-ΔCт^ method. Related samples were denoted as S (stomach), H (heat), G (gill), M (mid-gut), D (digestive gland) and SW (seawater).
(DOCX)

## References

[1] Tang K, Liu K, Jiao N, Zhang Y, Chen CT (2013) Functional metagenomic investigations of microbial communities in a shallow-sea hydrothermal system. PLOS ONE 8:e72958 10.1371/journal.pone.0072958 23940820 PMC3735525

[2] Martin W, Baross J, Kelley D, Russell MJ (2008) Hydrothermal vents and the origin of life. Nat Rev Microbiol 6: 805–814. 10.1038/nrmicro1991 18820700

[3] Dubilier N, Bergin C, Lott C (2008) Symbiotic diversity in marine animals: the art of harnessing chemosynthesis. Nat Rev Microbiol 6: 725–740. 10.1038/nrmicro1992 18794911

[4] Watsuji TO, Yamamoto A, Motoki K, Ueda K, Hada E, Takaki Y, et al (2015) Molecular evidence of digestion and absorption of epibiotic bacterial community by deep-sea crab *Shinkaia crosnieri*. ISME J 9: 821–831. 10.1038/ismej.2014.178 25314318 PMC4817695

[5] Durand L, Zbinden M, Cueff-Gauchard V, Duperron S, Roussel EG, Shillito B, et al (2010) Microbial diversity associated with the hydrothermal shrimp *Rimicaris exoculata* gut and occurrence of a resident microbial community. FEMS Microbiol Ecol 71: 291–303. 10.1111/j.1574-6941.2009.00806.x 19951370

[6] Jan C, Petersen JM, Werner J, Teeling H, Huang S, Glöckner FO, et al (2014) The gill chamber epibiosis of deep-sea shrimp *Rimicaris exoculata*: an in-depth metagenomic investigation and discovery of Zetaproteobacteria. Environ Microbiol 16: 2723–2738. 10.1111/1462-2920.12406 24447589

[7] Zbinden MI, Shillito B, Le Bris N, de Villardi de Montlaur C, Roussel E, Guyot F, et al (2008) New insights on the metabolic diversity among the epibiotic microbial community of the hydrothermal shrimp *Rimicaris exoculata*. J Exp Mar Bio Ecol 359:131–140.

[8] Goffredi SK, Jones WJ, Erhlich H, Springer A, Vrijenhoek RC (2008) Epibiotic bacteria associated with the recently discovered Yeti crab, *Kiwa hirsuta*. Environ Microbiol 10: 2623–2634. 10.1111/j.1462-2920.2008.01684.x 18564185

[9] Goffredi SK (2010) Indigenous ectosymbiotic bacteria associated with diverse hydrothermal vent invertebrates. Environ Microbiol Rep 2: 479–488. 10.1111/j.1758-2229.2010.00136.x 23766219

[10] Tsuchida S, Suzuki Y, Fujiwara Y, Kawato M, Uematsu K, Yamanaka T, et al (2011) Epibiotic association between filamentous bacteria and the vent-associated galatheid crab, *Shinkaia crosnieri* (Decapoda: Anomura). J Mar Biol Assoc U K 91:23–32.

[11] Ponsard J, Cambon-Bonavita MA, Zbinden M, Lepoint G, Joassin A, Corbari L, et al (2013) Inorganic carbon fixation by chemosynthetic ectosymbionts and nutritional transfers to the hydrothermal vent host-shrimp *Rimicaris exoculata*. ISME J 7:96–109. 10.1038/ismej.2012.87 22914596 PMC3526180

[12] Polz MF, Robinson JJ, Cavanaugh CM, Van Dover CL (1998) Trophic ecology of massive shrimp aggregations at a Mid-Atlantic Ridge hydrothermal vent site. Limnol Oceanogr 43:1631–1638.

[13] Jeng MS, Ng NK, Ng PKL (2004) Feeding behaviour: hydrothermal vent crabs feast on sea ‘snow’. Nature 432:969 15616550 10.1038/432969a

[14] Wang TW, Chan TY, Chan BKK (2014) Trophic relationships of hydrothermal vent and non-vent communities in the upper sublittoral and upper bathyal zones off Kueishan Island, Taiwan: a combined morphological, gut content analysis and stable isotope approach. Mar Biol 161: 2447–2463.

[15] Tang SL, Hong MJ, Liao MH, Jane WN, Chiang PW, Chen CB, et al (2011) Bacteria associated with an encrusting sponge (*Terpios hoshinota*) and the corals partially covered by the sponge. Environ Microbiol 13:1179–91. 10.1111/j.1462-2920.2010.02418.x 21265978

[16] Chen CP, Tseng CH, Tang SL (2011) The dynamics of microbial partnerships in the coral *Isopora palifera*. ISME J 5:728–740. 10.1038/ismej.2010.151 20962876 PMC3105734

[17] Jorgensen SL, Hannisdal B, Lanzén A, Baumberger T, Flesland K, Fonseca R, et al (2012) Correlating microbial community profiles with geochemical data in highly stratified sediments from the Arctic Mid-Ocean Ridge. Proc Natl Acad Sci U S A 109:2846–2855. 10.1073/pnas.1207574109 PMC3479504 23027979

[18] Edgar RC, Haas BJ, Clemente JC, Quince C, Knight R (2011) UCHIME improves sensitivity and speed of chimera detection. Bioinformatics 27:2194–2200. 10.1093/bioinformatics/btr381 21700674 PMC3150044

[19] Edgar RC (2013) UPARSE: highly accurate OTU sequences from microbial amplicon reads. Nat Methods 10:996–998. 10.1038/nmeth.2604 23955772

[20] Hügler M, Petersen JM, Dubilier N, Imhoff JF, Sievert SM (2011) Pathways of carbon and energy metabolism of the epibiotic community associated with the deep-sea hydrothermal vent shrimp *Rimicaris exoculata*. PLOS ONE 6:e16018 10.1371/journal.pone.0016018 21249205 PMC3017555

[21] Campbell BJ, Engel AS, Porter ML, Takai K (2006) The versatile ε-proteobacteria: key players in sulphidic habitats. Nat Rev Microbiol 4:458–468. 16652138 10.1038/nrmicro1414

[22] Comeault A, Stevens CJ, Juniper SK (2010) Mixed photosynthetic-chemosynthetic diets in vent obligate macroinvertebrates at shallow hydrothermal vents on Volcano 1, South Tonga Arc-evidence from stable isotope and fatty acid analyses. Cah Biol Mar 51:351–359.

[23] Hu MYA, Hagen W, Jeng MS, Saborowski E (2012) Metabolic energy demand and food utilization of the hydrothermal vent crab *Xenograpsus testudinatus* (Crustacea: Brachyura). Aquatic Biol 15:11–25.

[24] Kuo FW. Preliminary investigation of the hydrothermal activities of Kueishantao Island. Dissertation, National Sun Yat-sen University. 2001. Available: http://handle.ncl.edu.tw/11296/ndltd/34467227286873776166

[25] Chen CTA, Zeng Z, Kuo FW, Yang TF, Wang BJ, Tu YY (2005) Tide-influenced acidic hydrothermal system offshore NE Taiwan. Chem Geol 224:69–81.

[26] Shao MF, Zhang T, Fang HHP (2010) Sulfur-driven autotrophic denitrification: diversity, biochemistry, and engineering applications. Appl Microbiol Biotechnol 88:1027–1042. 10.1007/s00253-010-2847-1 20809074

[27] Peng SH, Hung JJ, Hwang JS (2011) Bioaccumulation of trace metals in the submarine hydrothermal vent crab *Xenograpsus testudinatus* off Kueishan Island, Taiwan. Mar Pollut Bull 63:396–401. 10.1016/j.marpolbul.2011.05.013 21658729

[28] Regassa LB, Gasparich GE (2006) *Spiroplasmas*: evolutionary relationships and biodiversity. Front Biosci 11:2983–3002. 16720370 10.2741/2027

[29] Ho TW, Hwang JS, Cheung MK, Kwan HS (2015) Dietary analysis on the shallow-water hydrothermal vent crab *Xenograpsus testudinatus* using Illumina sequencing. Mar Biol 162:1787–1798.

[30] Eddy F, Powell A, Gregory S, Nunan LM, Lightner DV, Dyson PJ, et al (2007) A novel bacterial disease of the European shore crab, *Carcinus maenas* molecular pathology and epidemiology. Microbiology 153:2839–2849. 17768229 10.1099/mic.0.2007/008391-0

[31] Singh RP, Reddy CR (2014) Seaweed-microbial interactions: key functions of seaweed-associated bacteria. FEMS Microbiol Ecol 88:213–230. 10.1111/1574-6941.12297 24512602

[32] Buchan A, González JM, Moran MA (2005) Overview of the marine *Roseobacter* lineage. Appl Environ Microbiol 71:5665–5677. 16204474 10.1128/AEM.71.10.5665-5677.2005 PMC1265941

[33] Ziesche L, Bruns H, Dogs M, Wolter L, Mann F, Wagner-Döbler I, et al (2015) Homoserine lactones, methyl oligohydroxybutyrates, and other extracellular metabolites of macroalgae-associated bacteria of the *Roseobacter* clade: identification and functions. Chembiochem 16:2094–2107. 10.1002/cbic.201500189 26212108

[34] Farmer JJ, Janda JM, Brenner FW, Cameron DN, Birkhead KM (2005) Genus I. *Vibrio pacini* 1854 In: Brenner DJ, Kreig NR, Staley JT, eds. Bergey’s Manual of Systematic Bacteriology. Springer: New York, Vol 2, pp 494–546.

[35] Pruzzo C, Huq A, Colwell RR, Donelli G (2005) Pathogenic Vibrio species in the marine and estuarine environment In: Belkin S, Colwell RR, eds. Ocean and Health Pathogens in the Marine Environment. Springer: New York Pp 217–252.

[36] Gullian M, Thompson F, Rodriguez J (2004) Selection of probiotic bacteria and study of their immunostimulatory effect in *Penaeus vannamei*. Aquaculture 233: 1–14.

[37] Hasan NA, Grim CJ, Lipp EK, Rivera IN, Chun J, Haley BJ, et al (2015) Deep-sea hydrothermal vent bacteria related to human pathogenic *Vibrio* species. Proc Natl Acad Sci U S A 112: E2813–2819. 10.1073/pnas.1503928112 25964331 PMC4450432

[38] Nyholm SV, McFall-Ngai MJ (2004) The winnowing: establishing the squid-vibrio symbiosis. Nat Rev Microbiol 2:632–642. 15263898 10.1038/nrmicro957

[39] Chen CA, Keshavmurthy S (2009) Taiwan as a connective stepping-stone in the Kuroshio Triangle and the conservation of coral ecosystems under the impacts of climate change. Kuroshio Science 3:15–22.

[40] Namias J (1970) Macroscale variations in sea-surface temperatures in the North Pacific. J Geophys Res 75:565–582.

[41] Chu TY (1974) The fluctuations of the Kuroshio current in the eastern sea area of Taiwan. Acta Oceanogr Taiwan 4:1–12.

[42] Hsin YC, Wu CR, Shaw PT (2008) Spatial and temporal variations of the Kuroshio east of Taiwan. 1982–2005: A numerical study. J Geophys Res 113: C04002.

[43] Wu CR, Chang YL, Oey LY, Chang CWJ, Hsin YC (2008) Air-sea interaction between tropical cyclone Nari and Kuroshio. Geophys Res Lett 35:L12605.

