# Supplementary material for: Bacterial Community Associated with Organs of Shallow Hydrothermal Vent Crab Xenograpsus testudinatus near Kuishan Island, Taiwan
Source: PLoS One. 2016 Mar 2;11(3):e0150597. doi: 10.1371/journal.pone.0150597 (PMC4774926; doi:10.1371/journal.pone.0150597)
Supplement: S1 Fig — Operational taxonomic units (OTUs) were defined by a 3% cut off value in sequence dissimilarity. Related samples were denoted as S (stomach), H (heat), G (gill), M (mid-gut), D (digestive gland) and SW (seawater). (DOCX) [file pone.0150597.s001.docx]

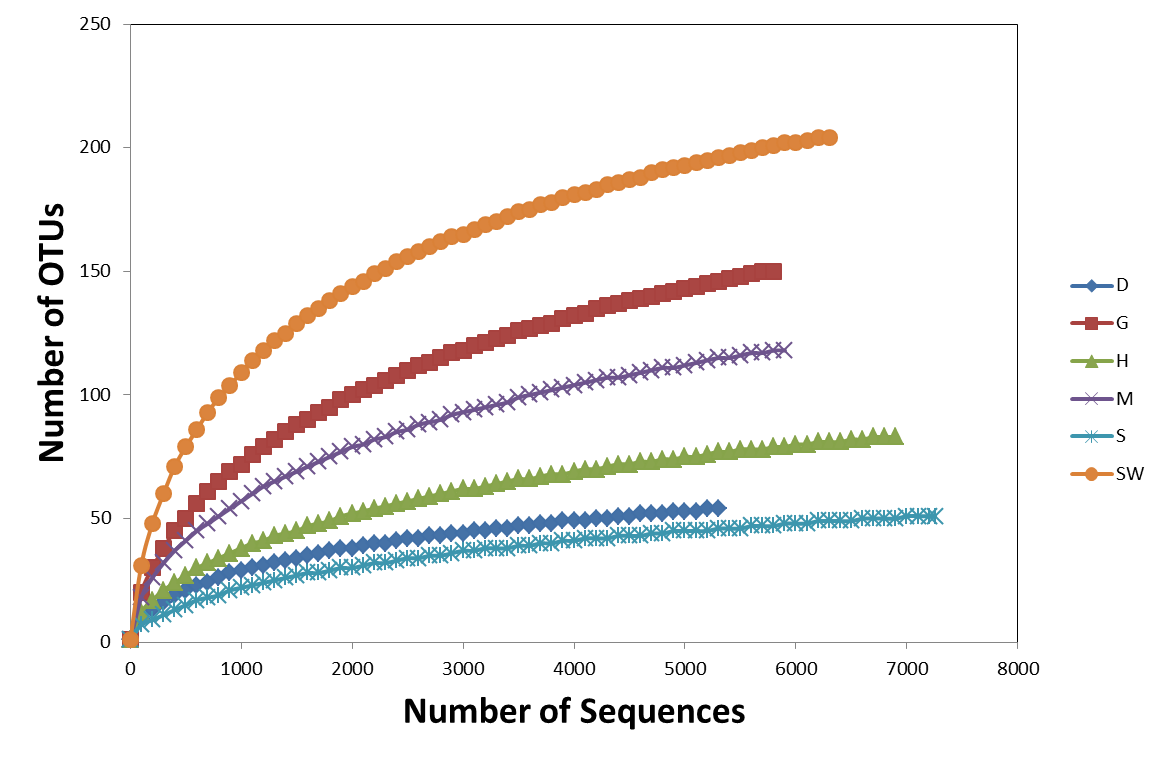


**Figure S1. The rarefaction curve of bacterial 16S rRNA genes in organs of *X. testudinatus* and seawater.**
